# Groundwater Quality in Agricultural Lands Near a Rapidly Urbanized Area, South China

**DOI:** 10.3390/ijerph18041783

**Published:** 2021-02-12

**Authors:** Lingxia Liu, Shihua Qi, Wenzhong Wang

**Affiliations:** 1 School of Environmental Studies, China University of Geosciences, Wuhan 430074, China; llingxia2004@163.com (L.L.); shihuaqi@cug.edu.cn (S.Q.); 2 Institute of Hydrogeology and Environmental Geology, Chinese Academy of Geological Sciences, Shijiazhuang 050061, China

**Keywords:** groundwater quality, agricultural areas, Pearl River Delta, wastewater infiltration, geogenic sources

## Abstract

Understanding the groundwater quality and its factors is a key issue in the context of the use and protection of groundwater resources in agricultural areas near urbanized areas. This study assessed the groundwater quality in agricultural areas in the Pearl River Delta (PRD) by a fuzzy synthetic evaluation method and determined the main factors controlling the groundwater quality by principal component analysis (PCA). Results showed that approximately 85% of groundwater sites in agricultural lands in the PRD were good-quality (drinkable). Drinkable groundwater was 95% and 80% in fissured aquifers and porous aquifers, respectively. Poor-quality groundwater in porous aquifers was controlled by four factors according to the PCA, including the seawater intrusion; the lateral recharge and irrigation of surface water and geogenic sources for As, Fe, NH_4_^+^, and Mn; the wastewater infiltration; and the geogenic sources for iodide. By contrast, another four factors, including the infiltration of wastewater and agricultural fertilizers, the geogenic sources for heavy metals, the geogenic sources for iodide, and the irrigation of contaminated river water, were responsible for the poor-quality groundwater in fissured aquifers. Therefore, in the future, the groundwater protection in agricultural lands in the PRD should be strengthened because the majority of groundwater in these areas was good-quality and suitable for drinking and agricultural purposes. In addition, poor-quality groundwater in agricultural lands in the PRD was a small proportion and negligible because the factors for poor-quality groundwater are complicated.

## 1. Introduction

Groundwater resources are important for agricultural purposes and rural residents in agricultural areas [1,2,3], and the groundwater quality is a key issue for the management of groundwater resources in these areas. For example, groundwater is often used for agricultural irrigation in agricultural lands in the Pearl River Delta (PRD), south China, because it is free of charge and convenient [4]. Thus, it is meaningful to understand the status of groundwater quality and the main factors controlling groundwater quality for groundwater management in agricultural areas in the PRD. To date, the status of groundwater quality in urbanized areas and peri-urban areas in the PRD was reported, but that in agricultural areas has received little attention [5].

Generally, the groundwater quality in agricultural areas is sensitive to the contaminants originating from agricultural chemicals, such as nitrate and chloride [1,6,7]. For instance, nitrogen fertilizers are used to increase crop yields and result in groundwater nitrate contamination because of the leaching of nitrate from farmland [8]. In the PRD, the groundwater quality in agricultural areas is not only affected by agricultural activities but may also be influenced by urbanization and industrialization, because agricultural lands are sometimes near urbanized areas [9,10]. Nowadays, factors controlling groundwater quality in agricultural areas in the PRD are also little known.

The aims of this study are to investigate the groundwater quality in agricultural lands in the PRD, and to analyze the main factors controlling groundwater quality by principal component analysis (PCA). The results would be in favor of the management of groundwater resources in agricultural areas near urbanized areas.

## 2. Study Area

**Figure 1:**
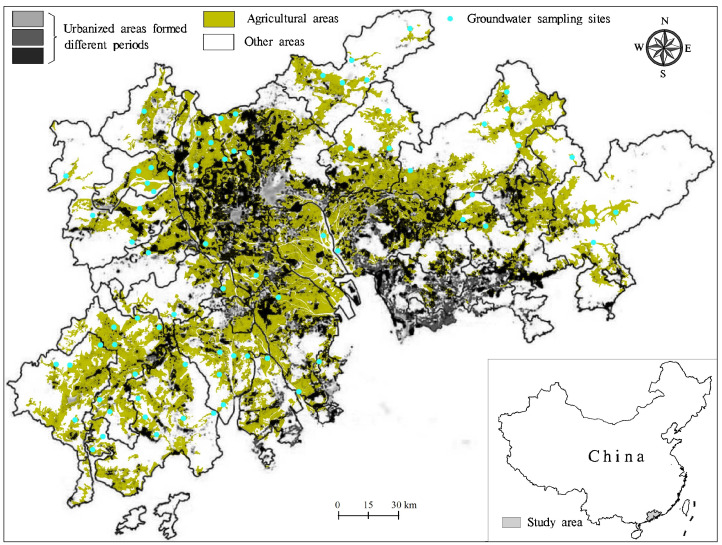
Groundwater sampling sites in agricultural areas in the Pearl River Delta (original data from [11]).

The PRD covers a total area of 41,698 km^2^ in the south-central part of Guangdong Province, China. It is within longitudes of 111°59′–115°25′ and latitudes of 21°17′–23°55′ (Figure 1). The west, north, and east of the PRD are surrounded by hills, while the south is adjacent to the South China Sea. It is a compound delta formed by the deposition of sediments from Xijiang River, Beijiang River, and Dongjiang River. As a result, a plain is formed in the center and south. The PRD is characterized by a marine monsoonal climate, and the wet season is from April to September.

The expansion of construction land in the PRD has been rapid since the 1980s. Urbanized areas (UA) in the PRD reached more than 6800 km^2^ in 2006, approximately 1.5 times that in 1998 [12]. By contrast, agricultural lands in the PRD covered a total area of 1.3 × 10^4^ km^2^ in 2006, and have decreased by a total area of approximately 1 × 10^3^ km^2^ from 1998 because of the increased urbanized areas [11]. Rice and vegetables are dominant plants in these agricultural lands, and approximately 2 × 10^8^ m^3^ groundwater has been used for agriculture irrigation in 2006 [13]. Note that some new landfills have been formed in agricultural areas during urbanization [14].

The PRD plain has been developed under the river–sea interactions since the late Quaternary. Quaternary deposits cover the central and southern parts of the PRD and compose the PRD plain where the vast majority of agricultural lands are distributed. Quaternary deposits are composed of four stratigraphic units including two marine units and two terrestrial units [12]. The younger terrestrial unit can be sandy fluvial deposits or clayey silt and become a local aquifer, and groundwater for agricultural irrigation in the PRD plain is mainly from this porous aquifer (Figures S1 and S2). The porous aquifer in coastal areas is often intruded by seawater. The fissured aquifer is distributed in hilly areas where there are fewer agricultural lands, and bedrocks ranging in age from Cambrian to Tertiary crop out within hilly areas [15].

## 3. Materials and Methods

### 3.1. Sampling and Analytical Techniques

In this study, 73 groundwater samples were collected from agricultural areas in the PRD during 2006. Among them, 51 and 22 samples were collected from the porous aquifer and fissured aquifer, respectively. In order to ensure that the groundwater samples were representative of the in situ conditions, samples were collected after purging at least 3 borehole volumes. In addition, samples were collected below the water table at a depth of 50 cm by a stainless steel sampler. Two 250 mL polyethylene bottles were used to store groundwater for the analysis of trace elements and other inorganic chemicals. One bottle used for trace elements analysis was acidified with nitric acid to a pH of less than 2. All the samples were stored at 4 °C until the laboratory procedures could be performed. All the samples were analyzed within 14 days after collection. A multi-parameter instrument was previously calibrated and used to analyze the redox potential (Eh), pH, and dissolved oxygen (DO) in situ. Inductively coupled plasma mass spectrometry (Agilent 7500ce ICP-MS, Tokyo, Japan) was used for the determination of concentrations of metals and trace elements (K^+^, Na^+^, Ca^2+^, Mg^2+^, Fe, Mn, Cu, Zn, As, Pb, Hg). The total dissolved solids (TDS) was determined by a gravimetric method. HCO_3_^−^ was determined by a titration method. Ion chromatography (Shimadzu LC-10ADvp, Japan) was used for the determination of concentrations of other anions (NO_3_^−^, SO_4_^2−^, Cl^−^, I^−^) and NH_4_^+^ [5]. The relative errors of inorganic parameters were <±6%. Note that the hydrochemical dataset in this study is the same one used in Zhang et al. [5]. This hydrochemical dataset is available for both this study and Zhang et al. [5]. Unlike Zhang et al. [5] who focused on the groundwater quality in urbanized areas and peri-urban areas in the PRD by using this hydrochemical dataset, this study focuses on the groundwater quality in agricultural areas.

### 3.2. Water Quality Assessment and Principal Component Analysis

Groundwater quality in agricultural lands in the PRD was assessed by a fuzzy synthetic evaluation (FSE) method. The details related to the FSE method are in supporting information (SI) [5,16]. Principal component analysis (PCA) is a powerful tool for analyzing hydrochemical data sets [17], as well as reducing a large number of variables to a small number of principal components (PCs) by linearly combining measurements made on the original variables [18]. This multi-step approach has been applied successfully to extract related variables and infer the underlying natural and/or anthropogenic processes that control the groundwater chemistry [19]. In this study, PCA was used to extract the PCs from groundwater chemical data sets that included 21 physico-chemical variables. Results of Bartlett’s test showed a significant difference between the correlation coefficient matrix and identity matrix and were suitable for the PCA (Table S1). Rotation of the PCs was conducted using the Varimax method, and PCs with eigenvalues >1 were retained for analyses. The terms “strong,” “moderate,” and “weak” (as applied to PC loadings) referred to the absolute loading values of >0.75, 0.75–0.5, and 0.5–0.3, respectively [10].

### 3.3. Fuzzy Synthetic Evaluation (FSE) Method

In this study, the fuzzy membership function was used to assess groundwater quality according to the groundwater quality standards of China (Table S2) [20]. The details of this FSE method are reported by Zhang et al. [5] as follows: “To reduce the complexity of the model, the linear membership functions are used:

(1) $$r_{ij}=\left\{\begin{array}{c} 0,\;\left(C_{i}\leq S_{ij-1}\;\mathrm{or}\;C_{i}\geq S_{ij-1}\right)\\ \frac{C_{i}-S_{ij-1}}{S_{ij}-S_{ij-1}},\;(S_{ij-1}<C_{i}<S_{ij})\\ \frac{S_{ij+1}-C_{i}}{S_{ij+1}-S_{ij}},\;\left(S_{ij}<C_{i}<S_{ij+1}\right)\\ 1,\;\left(C_{i}=S_{i}\right) \end{array}\right.$$

where r*_ij_* indicates the fuzzy membership of indicator *i* to class *j*, every indicator is characterized by five classes (I, II, III, IV, V) according to the groundwater quality standards of China [20], C*_i_* stands for the analytical value of groundwater quality indicator *i*, S*_ij_* stands for the allowable value of groundwater quality indicator. The fuzzy membership matrix R consists of groundwater quality indicators and classes.

The weight of groundwater quality indicator is expressed as:

(2) $$W_{i}=\frac{C_{i}}{S_{i}}$$

where W*_i_* is the weight of groundwater quality indicator *i*, C*_i_* is the analytical value of groundwater quality indicator *i*, S*_i_* is the arithmetic mean of allowable values of each class. The normalized weight of each indicator is calculated by the formula:

(3) $$a_{i}=\frac{C_{i}}{S_{i}}/\overset{m}{\underset{i=1}{\sum }}\frac{C_{i}}{S_{i}}=W_{i}/\overset{n}{\underset{i=1}{\sum }}W_{i}$$

where a*_i_* is the normalized weight of indicator *i*, W*_i_* is the sum of weight to all groundwater quality indicators. The fuzzy A consists of weight of each groundwater quality indicator.

The water quality assessment by fuzzy membership is based on the matrix B:

(4) B=A×R

The fuzzy B is the matrix of membership to each groundwater quality class. The groundwater sample is classified to the class with the maximum membership.

## 4. Results and Discussion

### 4.1. Groundwater Quality in Agricultural Lands in the PRD

**Figure 2:**
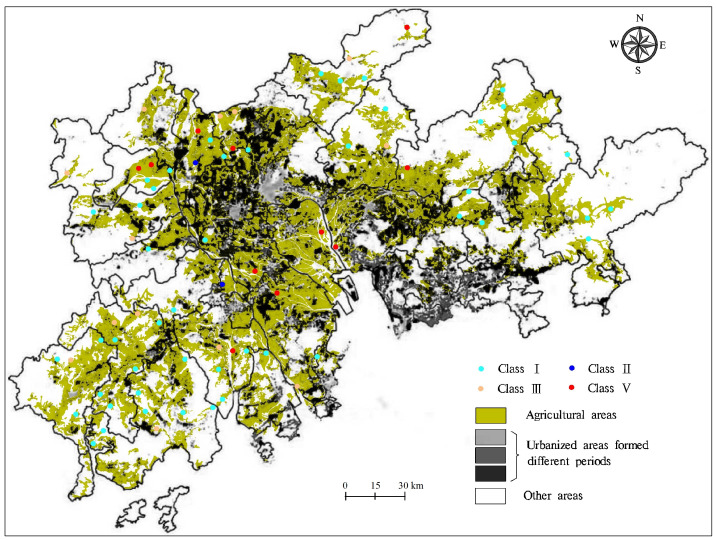
Spatial distribution of groundwater quality in agricultural areas in the Pearl River Delta.

The groundwater quality in agricultural lands in the PRD was assessed by the FSE method and classified into five classes according to the standards for groundwater quality of China [20]. In this study, indicators for groundwater quality assessment include 14 chemicals (Table S2), while the other seven chemicals (e.g., K, Ca, and Mg) are not included, because of the absence of standards of China. In addition, the allowable limits for these indicators in drinking water of the World Health Organization and United States Environmental Protection Agency are also shown in Table S2 [21,22]. As shown in Figure 2, groundwater quality in agricultural areas in the PRD was grouped into four classes; the contributions of classes I, II, III, and V were 64.4%, 2.7%, 17.8%, and 15.1%, respectively; and about 85% of groundwater samples were good-quality and drinkable (classes I, II, and III). As seen in Table 1, the contributions of classes I, II, III, and V for groundwater quality in porous aquifers in agricultural areas were 60.8%, 2.0%, 17.6%, and 19.6%, respectively, and approximately 80% of groundwater samples in porous aquifers in agricultural areas were drinkable. By contrast, groundwater quality in fissured aquifers in agricultural lands was also grouped into I, II, III, and V classes, accounting for 72.8%, 4.5%, 18.2%, and 4.5%, respectively, and approximately 95% of groundwater samples in fissured aquifers in agricultural lands were suitable for drinking (Table 1).

In this study, indicators resulting in poor quality (class V) for groundwater were investigated, and the proportion of groundwater samples with concentrations of one indicator exceeding the allowable value (class III in Table S2) (PEV) is shown in Table 1. In agricultural lands of the PRD, 10 indicators including Na^+^, Fe, Mn, As, Pb, TDS, NH_4_^+^, NO_3_^−^, Cl^−^, and I^−^ had PEV > 0 (Table 1). Similarly, in porous aquifers in agricultural areas, these 10 indicators also showed PEVs above zero, while others had zero PEVs (Table 1). Thus, these 10 indicators including Na^+^, Fe, Mn, As, Pb, TDS, NH_4_^+^, NO_3_^−^, Cl^−^, and I^−^ were impact indicators for poor-quality groundwater in porous aquifers in agricultural areas. By contrast, in fissured aquifers in agricultural areas, only five indicators including Fe, Mn, Pb, NO_3_^−^, and I^−^ had PEV > 0 (Table 1); as a consequence, these five indicators were impact indicators for poor-quality groundwater in fissured aquifers in agricultural areas.

### 4.2. Factors Controlling Groundwater Quality in Porous Aquifers in Agricultural Lands

A seven-factor model was extracted from the groundwater chemical data sets in porous aquifers in agricultural lands in the PRD by the PCA, and the cumulative variance of the seven PCs was 82.2% (Table 2).

Three impact indicators including Na^+^, Cl^−^, and TDS with positive loadings were in the same PC in porous aquifers (Table 2). This indicates that high concentrations of Na^+^, Cl^−^, and TDS in porous aquifers in agricultural lands may originate from the infiltration of sewage and seawater intrusion, because the sewage irrigation often occurs in agricultural lands and the seawater intrusion often occurs in porous aquifers in coastal areas in the PRD [4,5,12]. Moreover, the sewage in the PRD and seawater were characterized by much higher concentrations of Na^+^, Cl^−^, and TDS in comparison with those in porous aquifers in agricultural lands in the PRD (Table 3) [9,14,23]. However, PC1 also had a strong positive loading with Mg^2+^. Sewages in the PRD are generally characterized by high concentrations of Na^+^, Cl^−^, and TDS but not Mg^2+^ [11]. For example, Zhang et al. [14] reported that the mean concentrations of Na^+^, Cl^−^, and TDS in sewage-contaminated surface water in the PRD were 58.6, 64.2, and 489 mg/L, respectively, significantly higher than those in porous aquifers in agricultural lands in the PRD (Table 3). By contrast, the mean concentration of Mg^2+^ in sewage-contaminated surface water in the PRD was 6.9 mg/L [14], significantly lower than that in porous aquifers in agricultural lands in the PRD (Table 3). Thus, PC1 does not represent the infiltration of sewage, and the high concentrations of Na^+^, Cl^−^, and TDS in porous aquifers in agricultural lands were not attributed to the infiltration of sewage. On the other hand, the seawater not only shows high concentrations of Na^+^, Cl^−^, and TDS, but is also characterized by a high concentration of Mg^2+^ [12]. Therefore, PC1 represents the seawater intrusion, and the high concentrations of Na^+^, Cl^−^, and TDS in porous aquifers in agricultural lands in the PRD are likely ascribed to the seawater intrusion.

PC2 had strong positive loadings with impact indicators of As, Fe, and NH_4_^+^, and a positive loading with an impact indicator of Mn (Table 2). This indicates that high concentrations of As, Fe, NH_4_^+^, and Mn in porous aquifers in agricultural lands in the PRD originate from the same source or have similar geochemical behaviors. Huang et al. [15] reported that high concentrations of As and Fe in groundwater in porous aquifers in the PRD were mainly driven by reduction reactions in Fe/As-rich sediments resulting from the mineralization of organic matter and the formation of the reducing environment, because marine sediments in the PRD commonly enrich organic matter [24]. Jiao et al. [24] also reported that the mineralization of organic nitrogen in the overlying Holocene–Pleistocene aquitards in the PRD is mainly responsible for the high levels of NH_4_^+^ in groundwater in porous aquifers in the PRD, because these aquitards contain abundant organic nitrogen and are characterized by anoxic environments, which convert it to NH_4_^+^. In addition, Hou et al. [18] reported that the high concentration of Mn in groundwater in porous aquifers in the PRD was mainly attributed to the decomposition of organic matter and reduction in Fe (hydr)oxides in sediments with reducing condition. Thus, the natural mineralization of organic nitrogen in the overlying Holocene–Pleistocene aquitards, resulting in the reduction of As, Mn, and Fe (hydr)oxides in sediments, and finally leading to the co-release of As, Mn, Fe, and NH_4_^+^ from sediments, is likely to be responsible for the high levels of As, Fe, NH_4_^+^, and Mn in porous aquifers in agricultural lands in the PRD. On the other hand, high levels of As (>0.01 mg/L), Fe (>0.3 mg/L), NH_4_^+^ (>0.5 mg/L as N), and Mn (>0.1 mg/L) in contaminated surface water in the PRD often occur [4,14,18,23]. For instance, Huang et al. [9] reported that the mean concentrations of As and Fe in surface water in the PRD were 5.6 and 1.05 mg/L, respectively, approximately two times those in groundwater in porous aquifers in agricultural lands (Table 3). Similarly, Zhang et al. [14] reported that the mean concentration of NH_4_^+^ in surface water in the PRD was 11.47 mg/L, about eight times that in groundwater in porous aquifers in agricultural lands (Table 3). In addition, it is known that the irrigation with contaminated surface water for agricultural lands in the PRD often occurs, and the lateral recharge of surface water for groundwater in porous aquifers in the PRD also sometimes occurs [4,15,23]. Therefore, the lateral recharge and irrigation of contaminated surface water is probably another important factor resulting in high concentrations of As, Fe, NH_4_^+^, and Mn in groundwater in porous aquifers in agricultural lands in the PRD. As a consequence, PC2 is indicative of the lateral recharge and irrigation of surface water and geogenic sources for As, Fe, NH_4_^+^, and Mn. Correspondingly, high concentrations of As, Fe, NH_4_^+^, and Mn in porous aquifers in agricultural lands in the PRD are ascribed to the natural mineralization of organic nitrogen in aquitards, the reduction in As, Mn, and Fe (hydr)oxides in sediments, and the lateral recharge and irrigation of contaminated surface water.

PC4 showed strong positive loadings with impact indicators of Pb and NO_3_^−^ (Table 2). Huang et al. [4] reported that many small factories such as paper mills were near rivers and directly discharged the wastewater containing high levels of Pb into the rivers without treatment, and resulted in contaminated surface water with high concentrations of Pb. Meanwhile, the contaminated surface water was often used to irrigate the nearby agricultural lands. Similarly, Zhang et al. [5] also reported that the high level of Pb in groundwater in the PRD was mainly due to the infiltration of wastewater. As a consequence, the high concentration of groundwater Pb in porous aquifers in agricultural lands in the PRD is mainly ascribed to the infiltration of wastewater. On the other hand, Zhang et al. [14] also reported that the high level of NO_3_^−^ in porous aquifers originated mainly from the wastewater leakage of township–village enterprises during the industrialization. Therefore, PC4 is indicative of the wastewater infiltration, and the high levels of groundwater Pb and NO_3_^−^ in porous aquifers in agricultural lands in the PRD are attributed to the wastewater infiltration.

PC6 had strong positive loadings with Hg and the impact indicator of I^−^ (Table 2). Huang et al. [15] reported that the infiltration of industrial wastewater was responsible for the high level (>1 µg/L) of Hg in groundwater in the PRD. However, groundwater Hg in porous aquifers in agricultural lands in the PRD has shown low levels, and the maximum concentration was only 0.3 µg/L. Thus, we speculate that groundwater Hg in porous aquifers in agricultural lands in the PRD is a geogenic source, not anthropogenic source. On the other hand, Huang et al. [10] reported that the high level of I^−^ in porous aquifers in the PRD was mainly due to the reductive dissolution of iodine-loaded Fe (oxy)hydroxides and decomposition of iodine-rich organic matter in sediments. Correspondingly, the concentration of groundwater I^−^ in porous aquifers in agricultural lands in the PRD was as high as 0.59 mg/L (Table 3), much higher than that in contaminated surface water (<0.2 mg/L) in the PRD [10]. Therefore, the high level of groundwater I^−^ in porous aquifers in agricultural lands in the PRD was mainly attributed to the naturally reductive dissolution of iodine-loaded Fe (oxy)hydroxides and decomposition of iodine-rich organic matter. As a consequence, PC6 represents geogenic sources for Hg and I^−^.

### 4.3. Factors Controlling Groundwater Quality in Fissured Aquifers in Agricultural Lands

The groundwater chemistry in fissured aquifers in agricultural lands in the PRD was also controlled by a seven-factor model according to the PCA, and the cumulative variance of the seven PCs was 82.7% (Table 4).

PC1 explained 21.7% of the total variance, with strong positive loadings with Cl^−^, Na^+^, Mg^2+^, and impact indicators of Mn and NO_3_^−^ (Table 4). Generally, high levels of Cl^−^, Na^+^, and Mg^2+^ in groundwater in fissured aquifers in the PRD probably originated from water–rock interactions and the infiltration of agricultural fertilizers and wastewater [12]. However, anthropogenic sources, e.g., domestic sewage, industrial wastewater, and agricultural fertilizers, were commonly responsible for the high concentration of groundwater NO_3_^−^ in fissured aquifers in agricultural lands in the PRD [14]. Thus, the natural factors such as water–rock interactions were excluded out of PC1. On the other hand, groundwater with a high level of Mn sometimes occurred in agricultural lands near contaminated rivers, because contaminated river waters often enriched Mn and the irrigation of river water for agricultural lands often occurred in the PRD [18]. Therefore, PC1 represents the infiltration of wastewater and agricultural fertilizers, and high levels of Mn and NO_3_^−^ in groundwater in fissured aquifers in agricultural lands in the PRD are attributed to the infiltration of wastewater and agricultural fertilizers.

PC3 showed strong positive loadings with Zn and an impact indicator of Fe, and a moderate positive loading with Cu (Table 4). Groundwater Zn and Cu in fissured aquifers in agricultural lands had very low concentrations in comparison with the allowable values of China [20]. Similarly, in the PRD, mean concentrations of Fe in porous aquifers in agricultural lands, as well as in surface water, were more than 10 times those in fissured aquifers in agricultural lands (Table 3) [9]. These indicate that groundwater Zn, Cu, and Fe in fissured aquifers in agricultural lands likely originate from geogenic sources but not anthropogenic sources, because of the low concentrations of Zn, Cu, and Fe in groundwater. On the other hand, PC3 also showed a weak negative loading with DO. This indicates that high levels of groundwater Fe in fissured aquifers in agricultural lands are probably ascribed to the reduction in Fe and its oxides in strata, because Fe-rich soils often occur in bedrock areas in the PRD [12]. Therefore, PC3 is indicative of the geogenic sources for heavy metals, and the reduction in Fe and its oxides in strata is likely responsible for the high concentration of groundwater Fe in fissured aquifers in agricultural lands.

PC5 explained 8.1% of the total variance, with strong positive loadings with NH_4_^+^ and an impact indicator of I^−^ (Table 4). Zhang et al. [14] reported that high concentrations of groundwater NH_4_^+^ in fissured aquifers can be mainly ascribed to the mineralization of organic nitrogen in carbon-rich strata. Similarly, the degradation of iodine-rich organic matter in carbonate-rich rocks, such as mudstone and shale, was also responsible for the occurrence of iodide-rich groundwater in fissured aquifers [10]. As a consequence, PC5 represents the geogenic sources for NH_4_^+^ and I^−^, and the high level of groundwater I^−^ in fissured aquifers in agricultural lands is because of the mineralization of iodine-rich organic matter in sedimentary rocks.

PC6 had a strong positive loading with the impact indicator of Pb (Table 4). Zhang et al. (2019) reported that high levels of groundwater Pb in fissured aquifers in the PRD originated from the infiltration of industrial wastewater, because almost high concentrations (>0.01 mg/L) of groundwater Pb were distributed in urbanized and peri-urban areas where factories are common. Similarly, poor-quality groundwater with a high level of Pb in fissured aquifers in agricultural lands (southwest of the PRD) was in the river network area and close to urbanized areas (Figure 2). It is known that rivers near urbanized areas were sometimes contaminated by the industrial wastewater containing high levels of heavy metals, and that agricultural lands near rivers were often irrigated with river waters in the PRD [4,11,23]. Thus, the irrigation of contaminated river water is likely to be responsible for the high levels of Pb in fissured aquifers in agricultural lands in the PRD. In addition, acidic groundwater was in favor of the mobility of Pb in fissured aquifers because of the low pH values in groundwater in fissured aquifers and the negative loading with pH in PC6 (Table 3 and Table 4). As a result, PC6 is indicative of the irrigation of contaminated river water, and the high concentration of groundwater Pb in fissured aquifers in agricultural lands in the PRD is attributed to the irrigation of contaminated river water and acidic conditions.

## 5. Conclusions

The groundwater quality in agricultural lands in the PRD was investigated according to 14 inorganic indicators. Approximately 85% of groundwater sites in agricultural lands in the PRD were good-quality and drinkable. The groundwater quality in fissured aquifers was better than that in porous aquifers because drinking groundwater was 95% and 80% in fissured aquifers and porous aquifers, respectively.

In agricultural areas in the PRD, poor-quality groundwater in porous aquifers was due to the high levels of Na^+^, Fe, Mn, As, Pb, TDS, NH_4_^+^, NO_3_^−^, Cl^−^, and I^−^. Four factors, including the seawater intrusion; the lateral recharge and irrigation of surface water and geogenic sources for As, Fe, NH_4_^+^, and Mn; the wastewater infiltration; and the geogenic sources for I^−^, are responsible for the poor-quality groundwater in porous aquifers in agricultural areas in the PRD. By contrast, poor-quality groundwater in fissured aquifers was because of the high concentrations of Fe, Mn, Pb, NO_3_^−^, and I^−^. The poor-quality groundwater in fissured aquifers in agricultural areas in the PRD was also controlled by four factors, including the infiltration of wastewater and agricultural fertilizers, the geogenic sources for heavy metals, the geogenic sources for I^−^, and the irrigation of contaminated river water.

Therefore, in the future, the groundwater protection in agricultural lands in the PRD should be strengthened because the majority of groundwater in these areas was of good quality and suitable for drinking and agricultural purposes. The use and exploitation of groundwater for agricultural irrigation should be encouraged; meanwhile, it will be better to avoid the use of surface water for agricultural irrigation because surface waters are often contaminated and showed poor-quality in the PRD. In addition, groundwater with good quality in agricultural lands in the PRD can be water sources for emergency supply when floods occur, because agricultural lands are near urban areas in the PRD.

## Figures and Tables

**Table 1:** Groundwater quality and proportions of groundwater samples with concentrations of one indicator exceeding the allowable value (PEVs) of indicators in agricultural lands in the Pearl River Delta.

Items	PEV ^a^														Classes				
	Mn	Fe	I^−^	NH_4_^+^	As	TDS	Pb	Na^+^	Cl^−^	NO_3_^−^	SO_4_^2−^	Zn	Cu	Hg	I	II	III	IV	V
PRD (%)	31.5	19.2	13.7	12.3	5.5	4.1	4.1	4.1	4.1	2.7	0	0	0	0	64.4	2.7	17.8	0	15.1
Porous aquifer (%)	39.2	25.5	15.7	17.6	7.8	5.9	2	5.9	5.9	2	0	0	0	0	60.8	2	17.6	0	19.6
Fissured aquifer (%)	13.6	4.5	9.1	0	0	0	9.1	0	0	4.5	0	0	0	0	72.8	4.5	18.2	0	4.5

^a^ PEV: Proportion of groundwater samples with concentrations of one indicator exceeding the allowable value (class III in Table S2).

**Table 2:** Principal component (PC) loadings for groundwater chemistry in porous aquifers in agricultural lands in the Pearl River Delta.

Items	PCs						
	PC1	PC2	PC3	PC4	PC5	PC6	PC7
Na^+^	***0.973***	0.112	0.090	−0.013	−0.015	0.024	0.072
Cl^−^	***0.966***	0.112	0.054	0.008	−0.033	0.016	0.063
Mg^2+^	***0.892***	0.177	0.323	−0.088	0.059	−0.040	0.078
TDS	***0.819***	0.256	0.463	−0.099	0.062	0.036	0.136
As	0.085	***0.889***	0.167	−0.127	0.015	0.029	−0.022
Fe	0.220	***0.874***	0.010	−0.102	−0.081	0.141	0.175
NH_4_^+^	0.377	***0.790***	−0.062	−0.127	0.001	0.166	0.139
Mn	−0.074	***0.537***	0.304	−0.025	−0.145	0.147	−0.384
Ca^2+^	0.165	0.335	***0.814***	−0.206	0.195	0.067	−0.005
SO_4_^2−^	0.315	−0.206	***0.808***	0.084	0.010	0.113	0.172
HCO_3_^−^	0.419	0.483	***0.591***	−0.340	0.199	0.034	0.049
pH	0.154	0.303	***0.571***	−0.492	0.050	−0.319	−0.048
K^+^	0.298	0.019	***0.558***	0.257	−0.224	0.084	0.537
Pb	−0.052	−0.027	−0.157	***0.816***	0.124	−0.132	−0.269
NO_3_^−^	−0.006	−0.335	0.078	***0.757***	−0.242	−0.096	0.275
Zn	−0.147	0.011	−0.123	0.152	***0.783***	−0.085	0.142
Cu	0.069	−0.211	0.231	−0.091	***0.769***	−0.071	0.110
Eh	−0.227	−0.160	−0.123	0.328	***−0.619***	−0.289	0.148
I^−^	−0.038	0.025	−0.012	−0.167	0.011	***0.820***	−0.114
Hg	0.072	0.305	0.112	0.044	−0.044	***0.808***	0.099
DO	−0.144	−0.155	−0.131	0.108	−0.223	0.041	***−0.805***
Eigenvalue	4.1	3.4	2.9	2.0	1.9	1.6	1.4
Explained variance (%)	19.4	16.0	13.8	9.3	8.9	7.9	6.8
Cumulative % of variance	19.4	35.4	49.2	58.6	67.5	75.4	82.2

Bold and italic numbers = maximum absolute PC loading of one parameter.

**Table 3:** Statistics of the concentrations of groundwater chemicals in agricultural lands in the Pearl River Delta.

Items	Statistics	pH	Eh	DO	K^+^	Na^+^	Ca^2+^	Mg^2+^	HCO_3_^−^	Cl^−^	SO_4_^2−^	NO_3_^−^	I^−^	Zn	Cu	Hg	Pb	NH_4_^+^	Fe	TDS	Mn	As
			mV	mg/L																		
PRD	Min	4.26	−200	1.35	0.4	1.0	1.6	−	3.1	2.8	−	0.1	−	−	−	−	−	−	−	17	−	−
	Max	7.55	298	7.42	56.5	802.5	99.2	118.5	459.0	1619.9	126.6	116.4	1.19	0.170	0.013	0.0009	0.046	40.0	11.13	3152	2.64	0.0300
	Mean	6.03	111	3.74	15.5	37.3	32.0	8.2	97.4	68.7	22.7	25.1	0.05	0.033	0.001	−	0.003	0.99	0.46	318	0.23	0.0022
Porous aquifer	Min	4.67	−30	1.35	1.0	1.6	2.0	0.5	6.1	3.5	−	0.8	−	−	−	−	−	−	−	43	−	−
	Max	7.55	298	7.40	56.5	802.5	98.5	118.5	459.0	1619.9	126.6	116.4	0.59	0.170	0.013	0.0003	0.015	40.0	11.13	3152	2.64	0.0300
	Mean	6.12	109	3.63	18.3	47.2	36.6	10.3	116.1	86.8	28.2	25.2	0.04	0.028	0.002	−	0.002	1.40	0.63	379	0.30	0.0029
Fissured aquifer	Min	4.26	−200	1.76	0.4	1.0	1.6	−	3.1	2.8	−	0.1	−	−	−	−	−	−	−	17	−	−
	Max	6.83	295	7.42	33.7	65.6	99.2	12.6	206.7	122.3	46.0	91.4	1.19	0.160	0.006	0.0009	0.046	0.20	0.36	518	0.61	0.0014
	Mean	5.81	115	4.01	9.1	14.5	21.3	3.3	53.9	26.7	9.9	24.9	0.06	0.045	0.001	−	0.004	0.05	0.06	176	0.07	0.0005

−: Below the detection limits.

**Table 4:** Principal component (PC) loadings for groundwater chemistry in fissured aquifers in agricultural lands in the Pearl River Delta.

Fissured Aquifer	PCs						
	PC1	PC2	PC3	PC4	PC5	PC6	PC7
Cl^−^	***0.929***	0.062	−0.060	−0.008	−0.110	−0.002	0.177
Na^+^	***0.881***	0.089	−0.050	−0.003	−0.137	−0.057	0.204
Mn	***0.797***	−0.352	0.057	0.139	0.191	0.086	0.030
NO_3_^−^	***0.796***	0.234	−0.117	0.043	0.067	0.322	0.110
Mg^2+^	***0.752***	0.052	−0.202	−0.216	0.069	−0.012	−0.058
HCO_3_^−^	0.017	***0.935***	0.016	−0.213	0.114	−0.121	−0.042
Ca^2+^	0.339	***0.887***	0.002	−0.031	0.184	−0.044	−0.035
TDS	0.662	***0.694***	−0.053	−0.098	0.072	−0.027	0.223
Eh	0.257	***−0.692***	−0.244	−0.058	−0.125	0.202	−0.166
Zn	−0.098	0.131	***0.849***	−0.212	0.041	−0.023	0.215
Fe	−0.188	−0.067	***0.848***	−0.008	0.025	−0.012	−0.148
Cu	−0.093	−0.010	***0.688***	0.463	−0.154	−0.058	−0.236
DO	−0.364	−0.207	***−0.382***	0.284	0.306	0.290	0.017
Hg	−0.022	−0.073	0.010	***0.809***	−0.029	−0.008	0.066
As	0.121	0.241	0.516	***−0.604***	−0.047	−0.151	−0.088
I^−^	−0.103	0.117	0.086	−0.111	***0.860***	−0.039	0.022
NH_4_^+^	0.231	0.411	−0.199	0.113	***0.798***	−0.052	−0.025
Pb	0.078	−0.141	−0.097	−0.065	−0.017	***0.935***	0.105
pH	−0.131	0.520	−0.027	−0.376	0.154	***−0.627***	0.000
K^+^	0.181	0.017	−0.030	−0.043	−0.078	0.250	***0.910***
SO_4_^2−^	0.370	0.120	−0.125	0.382	0.180	−0.259	***0.719***
Eigenvalue	4.6	3.4	2.5	1.8	1.7	1.7	1.7
Explained variance (%)	21.7	16.3	12.0	8.6	8.1	8.0	7.9
Cumulative % of variance	21.7	38.0	50.0	58.6	66.8	74.8	82.7

Bold and italic numbers = maximum absolute PC loading of one parameter.

## Data Availability

The datasets generated and/or analyzed during the current study are not publicly available.

## Supplementary Materials

The following are available online at https://www.mdpi.com/1660-4601/18/4/1783/s1, Supplementary Table S1. Bartlett’s test for the suitability of PCA in groundwater chemical data of porous and fissured aquifers; Supplementary Table S2. Standards for groundwater quality of China and drinking water quality of WHO and US EPA; Supplementary Figure S1. Groundwater sampling sites in various aquifers in the Pearl River Delta.
