# Supplementary material for: Groundwater Quality in Agricultural Lands Near a Rapidly Urbanized Area, South China
Source: Int J Environ Res Public Health. 2021 Feb 12;18(4):1783. doi: 10.3390/ijerph18041783 (PMC7917582; doi:10.3390/ijerph18041783)
Supplement: Supplementary file 1 [file ijerph-18-01783-s001.pdf]

International Journal of
Environmental Research and Public Health

MDPI

Supplementary Table S1. Bartlett's test for the suitability of PCA in groundwater chemical data of porous and fissured aquifers.

Items		Porous Aquifers	Fissured Aquifers
Bartlett's test of sphericity	Approximate chi-square	937.945	897.425
	Significance	0.000	0.000

Supplementary Table S2. Standards for groundwater quality of China and drinking water quality of WHO and US EPA.

Item	Class I	Class II	Class III	Allowable Limits of WHO	Allowable Limits of US EPA	Class IV	Class V
TDS (mg/L)	≤300	≤500	≤1000		≤500	≤2000	>2000
Cl^- (mg/L)	≤50	≤150	≤250		≤250	≤350	>350
NO_3^- (mg/L)	≤8.9	≤22.1	≤88.6	≤50	≤44.3	≤132.9	>132.9
Na^+ (mg/L)	≤100	≤150	≤200			≤400	>400
SO_4^{2-} (mg/L)	≤50	≤150	≤250		≤250	≤350	>350
Fe (mg/L)	≤0.1	≤0.2	≤0.3		≤0.3	≤2	>2
Mn (mg/L)	≤0.05	≤0.05	≤0.1		≤0.05	≤1.5	>1.5
NH_4^+ (mg/L)	≤0.026	≤0.13	≤0.64			≤1.93	>1.93
Zn (mg/L)	≤0.05	≤0.5	≤1		≤5	≤5	>5
Pb (μg/L)	≤5	≤5	≤10	≤10	≤15	≤100	>100
Cu (μg/L)	≤10	≤50	≤1000	≤2000	≤1000	≤1500	>1500
As (μg/L)	≤1	≤1	≤10	≤10	≤10	≤50	>50
I^- (μg/L)	≤40	≤40	≤80			≤500	>500
Hg (μg/L)	≤0.1	≤0.1	≤1	≤6	≤2	≤2	>2
Suitability	Drinking, Irrigation	Drinking, Irrigation	Drinking, Irrigation	Drinking, Irrigation	Drinking, Irrigation	Irrigation	Not suitable

Data from General administration of quality supervision inspection and quarantine of the people's republic of China [20], World Health Organization [22], and United States Environmental Protection Agency [21].

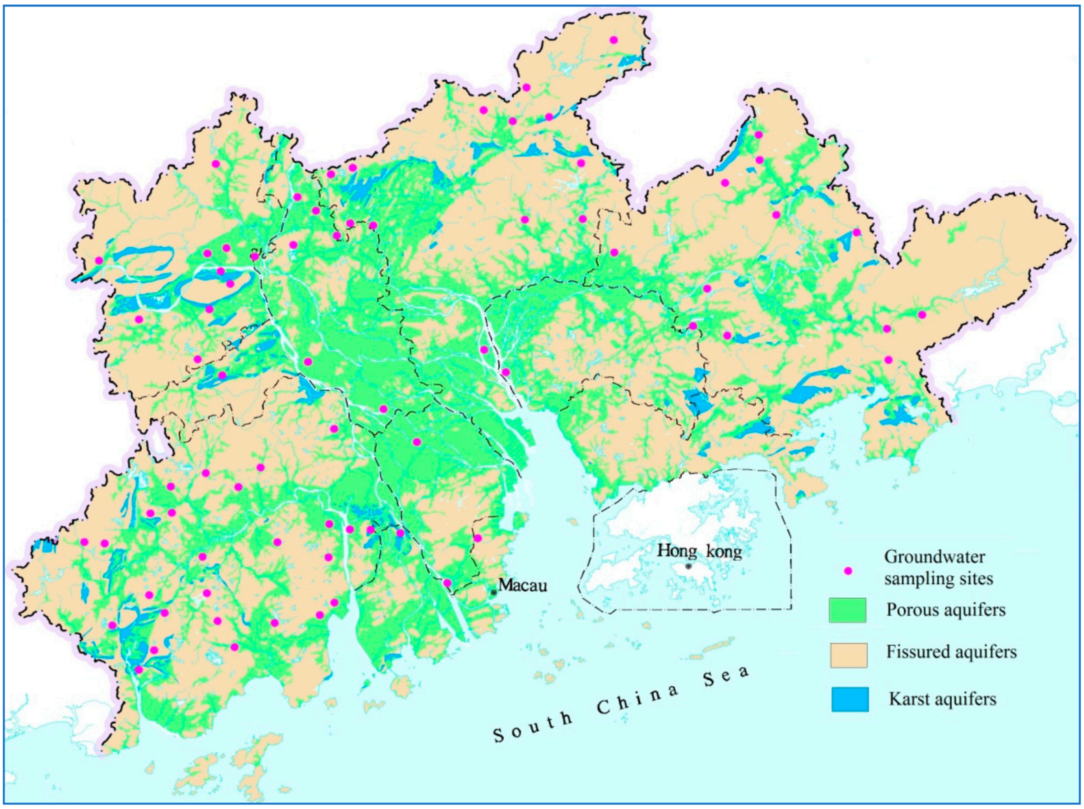

Supplementary Figure S1. Groundwater sampling sites in various aquifers in the Pearl River Delta.
